# Association of Ambient Air Pollution Exposure With Incident Glaucoma: 12-Year Evidence From the UK Biobank Cohort

**DOI:** 10.1167/iovs.65.12.22

**Published:** 2024-10-16

**Authors:** Zihan Sun, Kelsey V. Stuart, Robert N. Luben, Amy L. Auld, Nicholas G. Strouthidis, Peng T. Khaw, Hari Jayaram, Anthony P. Khawaja, Paul J. Foster, on behalf of the UK Biobank Eye and Vision Consortium

**Affiliations:** 1 NIHR Biomedical Research Centre at Moorfields Eye Hospital NHS Foundation Trust and UCL Institute of Ophthalmology, London, United Kingdom; 2 Glaucoma Service, Moorfields Eye Hospital NHS Foundation Trust, London, United Kingdom; 3 Discipline of Clinical Ophthalmology and Eye Health, University of Sydney, Sydney, New South Wales, Australia

**Keywords:** air pollution, glaucoma, fine particulate matter

## Abstract

**Purpose:**

Glaucoma is the leading cause of irreversible blindness worldwide. Despite growing concerns about air quality and its impact on ocular health, there remains a knowledge gap regarding the long-term association between air pollution and glaucoma risk. This study investigates the relationship between exposure to ambient air pollution and incidence of glaucoma.

**Methods:**

In this prospective study, we used land use regression models to estimate levels of various air pollutants, including fine particulate matter (PM_2.5_), PM_2.5 absorbance_, PM_2.5-10_, PM_10_, nitrogen dioxide (NO_2_), and nitrogen oxides (NO_x_). Incidents of glaucoma were ascertained through routinely collected hospital admission records. Multivariate Cox proportional hazards models were used to examine the associations between air pollution exposure and glaucoma incidence, adjusting for potential confounding sociodemographic, physical, and lifestyle factors.

**Results:**

Data from 481,113 participants were included. Over a median follow-up of 12.8 years, 9224 incident cases of glaucoma were identified. In the maximally adjusted model, per interquartile range increase in PM_2.5_ was associated with a 3% greater risk of developing glaucoma (hazard ratio [HR] = 1.03, 95% confidence interval [CI] = 1.00 to 1.06, *P* = 0.048). Participants in the highest quartile had a 10% increased risk of developing glaucoma compared to those in the lowest quartile (HR = 1.10, 95% CI = 1.03 to 1.17, *P* = 0.005).

**Conclusions:**

Higher levels of exposure to ambient air pollutants, particularly PM_2.5_, are associated with an increased risk of developing glaucoma. These results highlight the potential public health impact of ambient air pollution on glaucoma risk and underscore the urgent need for further research into targeted environmental interventions in this domain.

Air pollution poses a significant environmental threat to human health, with the World Health Organization (WHO) estimating 7 million deaths annually attributed to this hazard – comparable to other major health risks, such as smoking and unhealthy diets.^1^ Of particular concern among pollutants is particulate matter with an aerodynamic diameter less than 2.5 µm (PM_2.5_), known for its capacity to infiltrate not only the lungs but also the bloodstream. Over the past 2 decades, a substantial body of evidence has emerged, revealing the adverse health impacts of air pollution even at concentrations lower than previously recognized.^2^^–^^4^ In response to this evolving understanding, the WHO has revised the global air quality guidelines by halving the annual maximum level of PM_2.5_ from 10 µg/m^3^ (in the 2005 version) to 5 µg/m^3^ (in the 2021 version).^5^^,^^6^ This new guideline not only underscores the severity of the issue but also signals a shift in acknowledging the systemic impact of air pollution beyond traditionally associated respiratory concerns.

Glaucoma, a common age-related neurodegenerative disease, stands as the leading cause of irreversible blindness globally.^7^ Previous observations reported a 50% higher likelihood of glaucoma in urban residents compared to their rural counterparts, hinting that air pollution could be a potential risk factor for glaucoma.^8^ Moreover, numerous studies have shed light on the role of air pollution in triggering systemic inflammation, oxidative stress, and endothelial dysfunction – mechanisms implicated in the pathogenesis of various diseases, including glaucoma.^9^^–^^12^ This prompts the consideration of air pollution as a biologically plausible factor contributing to the development of glaucoma.

Several cross-sectional studies in the United Kingdom, Canada, and East Asia have explored the relationship between air pollution and glaucoma, consistently revealing a positive association between PM_2.5_ and a greater odds of prevalent glaucoma.^13^^–^^17^ As cross-sectional evidence provides only snapshots of relationships at a single time point, longitudinal data become essential for validating such findings. Recognizing the limited longitudinal evidence available,^18^^,^^19^ we aim to investigate the association between air pollution exposure and incident glaucoma cases in the UK Biobank. Furthermore, leveraging the genetic data available in the UK Biobank, we intend to explore potential gene-environment interactions by assessing whether the noted association is modified by a multitrait glaucoma polygenic risk score (PRS).

## Methods

### Study Participants

The UK Biobank is a large-scale community-based cohort of half a million UK residents registered with the National Health Service (NHS) and aged 37 to 73 years at enrollment. Initial assessment took place from 2006 to 2010 across 22 centers in the United Kingdom. The study was approved by the North West Multi-Centre Research Ethics Committee (reference no. 06/MRE08/65) and adheres to the Declaration of Helsinki. Overall study protocol is provided online in the public domain (https://www.ukbiobank.ac.uk/media/gnkeyh2q). Further details about the study can be found on the UK Biobank website at http://www.ukbiobank.ac.uk/. Details on the assessments of covariates, genotyping, and PRS^20^ are provided in the Supplementary eMethods.

### Exposures

For each participant, ambient air pollution estimates were generated from their residential addresses using a land use regression (LUR) model developed as part of the European Study of Cohorts for Air Pollution Effects (ESCAPE, http://www.escapeproject.eu/) project.^21^^,^^22^ These estimates included concentrations of particulate matter (PM) and nitrogen oxides, which are significant components of air pollution with known health impacts.

Particulate matter is categorized by its aerodynamic diameter: PM_10_ consists of particles with a diameter of 10 micrometers (µm) or less, capable of being inhaled into the lungs; PM_2.5_ refers to finer particles less than 2.5 µm in diameter, which are small enough to reach deep into the alveolar region and may potentially penetrate the respiratory barrier to enter the circulatory system^23^^–^^25^; PM_2.5-10_ represents the coarser fraction of particles between 2.5 and 10 µm. PM_2.5 absorbance_, which measures the light absorption (blackness) of PM_2.5_ filters, is indicative of elemental carbon typically emitted from combustion sources. Nitrogen oxides (NO_x_), a collective term for nitrogen dioxide (NO_2_) and nitric oxide (NO), are gaseous pollutants primarily resulting from traffic-related emissions and other combustion processes.

The LUR model utilized monitoring data collected from January 26, 2010, to January 18, 2011, to represent the annual average air pollution levels for the year 2010. According to ESCAPE protocol, estimates for PM from the models are considered reliable within a 400 km radius from Greater London – the designated monitoring area. Addresses outside this radius, where validation of accuracy was not available, were consequently assigned missing values for PM_10_, PM_2.5-10_, PM_2.5_, and PM_2.5 absorbance_ concentrations and were excluded in this analysis.

### Ascertainment of Glaucoma Cases and Study End Points

Incident glaucoma was identified through data linkage with hospital inpatient admission records, specifically the Hospital Episode Statistics (HES) for England, Scottish Morbidity Record (SMR) for Scotland, and the Patient Episode Database for Wales (PEDW). This includes data on admissions for overnight stays and day cases, but does not cover outpatient clinic attendances. Glaucoma cases were ascertained by either: (1) International Classification of Diseases (ICD) code for glaucoma (ICD 9th revision: 365 [365.1, 365.2, 365.3, 365.4, 365.5, 365.6, 365.8, and 365.9]; ICD 10th revision: H40 [H40.1, H40.2, H40.3, H40.4, H40.5, H40.6, H40.8, H40.9, H42.0, and H42.8]), or (2) an Office of Population Censuses and Surveys Classification of Interventions and Procedures, version 4 (OPCS-4) code for glaucoma-related procedures (C60.1 [trabeculectomy], C60.5 [insertion of a tube into the anterior chamber of the eye to assist drainage of aqueous humour], and C66.4 [laser photocoagulation of the ciliary body]).

As of our analysis in January 2024, the most recent hospital inpatient records were available up to October 31, 2022, for England, August 31, 2022, for Scotland, and May 31, 2022, for Wales. Death registry records were up to date through November 30, 2022, for all regions. Data on lost to follow-up, such as cases where an individual left the United Kingdom but did not withdraw consent for participation, were updated to May 2017. We chose October 31, 2022, as the cutoff date for data linkage to align with the most comprehensive and current data set from England, the largest constituent country in our study. The study baseline was set as January 1, 2010, to coincide with the period when air pollution levels were estimated to reflect the average level for the year of 2010. Time to incident glaucoma was calculated as the duration from the study baseline (January 1, 2010) to the first recorded instance of an ICD-coded glaucoma diagnosis or an OPCS-4-coded glaucoma procedure. The follow-up period was defined as the time from the study baseline (January 1, 2010) to the date of the last censored event – death, loss to follow-up, or the end of data linkage (October 31, 2022), whichever came first.

### Inclusion and Exclusion Criteria

We included participants who had given consent to the UK Biobank as of the data request date in January 2024. Participants were excluded if they met any of the following criteria: (1) a self-reported diagnosis of glaucoma, use of intraocular pressure (IOP)-lowering medications, or previous surgical or laser treatment in either eye at the initial assessment visit from 2006 to 2010; (2) OPCS-4-coded glaucoma procedures recorded at any point up to 1 year after the study baseline (January 1, 2010), that is, before December 31, 2010; (3) the first instance of any ICD-10 coded glaucoma diagnosis from primary care data, hospital inpatient data, death registry records, or self-reported medical condition codes before December 31, 2010; (4) a diagnosis of glaucoma suspect (ICD-10: H40.0) in hospital inpatient records before December 31, 2010; and (5) death or loss to follow-up on or before December 31, 2010. Additionally, participants with missing data on air pollution estimates were excluded from the analysis.

### Statistical Analysis

Descriptive statistics comprise counts (percentages) for categorical variables, and means ± standard deviations (SDs) or medians (interquartile ranges [IQRs]) for continuous variables. The choice between mean ± SD and median (IQR) presentations was made depending on the distribution of the variables. For investigating the associations between various air pollutants (PM_10_, PM_2.5-10_, PM_2.5_, PM_2.5 absorbance_, NO_2_, and NO_x_) and time to incident glaucoma, we estimated hazard ratios (HRs) using Cox proportional hazards. Analyses were conducted using both unadjusted model and two multivariable models, A and B. Model A accounted for age and sex, and model B further adjusted for ethnicity, the Townsend deprivation index, body mass index, and smoking status. To assess whether the relationship between air pollution exposures and the incident glaucoma is modified by the glaucoma PRS, we tested the significance of a multiplicative interaction term between air pollution and the genetic factor in the maximally adjusted model. The glaucoma PRS^20^ was included as a continuous variable in these models and was normalized with a mean of 0 and an SD of 1 for analyses. Statistical analyses were performed with R software (version 4.3.2; R Foundation for Statistical Computing, Vienna, Austria). *P* values less than 0.05 were considered statistically significant.

## Results

**Figure 1.**
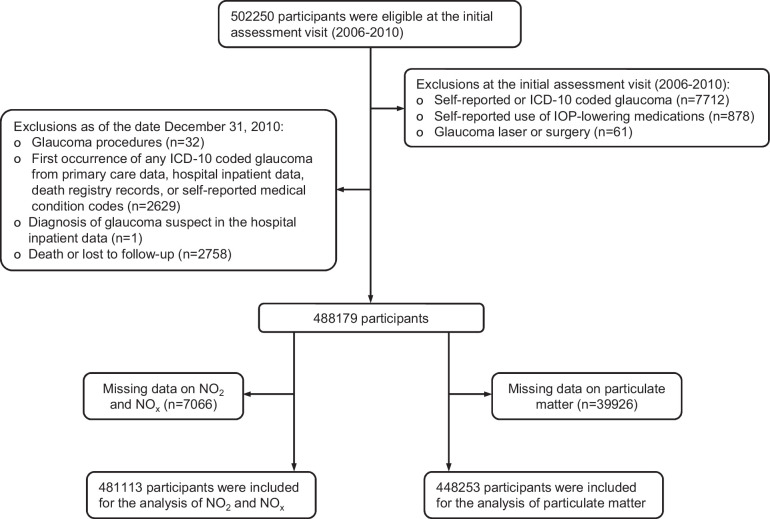
Flow diagram. The baseline for the study is established at the time when the air pollution levels were estimated, specifically in the year 2010. Accordingly, any events that occurred before December 31, 2010, were excluded. ICD, International Classification of Diseases; IOP, intraocular pressure; NO_2_, nitrogen dioxide; NO_x_, nitric oxides.

Of the 502,250 UK Biobank participants, 481,113 were included in the final analysis for NO_2_ and NO_x_, and 448,253 were included for analysis of PM_10_, PM_2.5-10_, PM_2.5_, and PM_2.5 absorbance_ (Fig. 1). Table 1 summarizes the characteristics of the study cohort and air pollution levels. Over a median follow-up of 12.8 years, approximately 1.9% to 2.0% of the participants developed incident glaucoma, corresponding to 9224 cases from the NO_2_/NO_x_ data set and 8999 cases from the PM data set.

**Table 1.** Characteristics of the Study Population and Air Pollution Levels

Characteristic	No	Mean ± SD, Median (IQR), or %	Range
Age at recruitment, y	481,113	56.42 ± 8.09	37.00 to 73.00
Age at study baseline, y	481,113	57.86 ± 8.11	38.13 to 75.80
Sex	481,113		
Female	263,024	54.67%	
Male	218,089	45.33%	
Ethnicity	481,113		
White	453,084	94.17%	
Asian	10,893	2.26%	
Black	7,527	1.57%	
Others/mixed/unknown	9,609	2.00%	
Townsend deprivation index	480,540	–2.16 (4.15)	–6.26 to 11.00
Duration of follow-up, y	481,113	12.83 (0)	1.00 to 12.83
No. of death	39,683	8.25%	
Smoking status	478,332		
Never	263,022	54.99%	
Previous	165,134	34.52%	
Current	50,176	10.49%	
Body mass index, kg/m^2^	478,262	27.42 ± 4.79	12.12 to 74.68
Air pollutants, µg/m³			
PM_2.5_	448,253	9.93 (1.27)	8.17 to 21.31
PM_2.5 absorbance_	448,253	1.13 (0.3)	0.83 to 4.60
PM_2.5-10_	448,253	6.11 (0.8)	5.57 to 12.82
PM_10_	448,253	16.24 ± 1.9	11.78 to 31.39
NO_2_	481,113	26.23 (9.8)	12.93 to 108.49
NO_x_	481,113	42.41 (16.4)	19.74 to 265.94

Categorical variables are presented as count and percentage. Continuous variables are displayed as mean ± SD or as median (IQR). The choice between mean ± SD and median (IQR) presentation is based on the normality of the variable distribution. Particulate matter definitions: (PM_2.5_) Finer particles with a diameter less than 2.5 µm; (PM_2.5 absorbance_) Measures light absorption (blackness) of PM_2.5_ filters, served as a proxy of elemental carbon typically emitted from combustion sources; (PM_10_) Particles with a diameter of 10 µm or less; (PM_2.5-10_) Coarse particulate fraction between 2.5 µm and 10 µm in diameter.

IQR, interquartile range; kg/m^2^, kilogram per square meter; µg/m³, microgram per cubic meter; NO_2_, nitrogen dioxide; NO_x_, nitrogen oxides; PM, particulate matter; SD, standard deviation.

For PM (Table 2), PM_2.5_ was significantly associated with the risk of glaucoma. Univariable analysis showed a 3% increase in glaucoma risk (HR = 1.03, 95% confidence interval [CI] = 1.01 to 1.06, *P* = 0.008) per IQR increment in PM_2.5_ concentration. This association remained statistically significant in multivariable analyses with an HR of 1.08 (95% CI = 1.06 to 1.11, *P* < 0.001) when adjusted for age and sex in model A, and an HR of 1.03 (95% CI = 1.00 to 1.06, *P* = 0.048) in the maximally adjusted multivariable model B, which included adjustments for age, sex, ethnicity, Townsend deprivation index, body mass index, and smoking status. In the quartile-based analysis, a discernible 10% increase in risk (HR = 1.10, 95% CI = 1.03 to 1.17, *P* = 0.005) was observed in the highest exposure quartile (Q4: 10.57 µg/m³ to 21.31 µg/m³) compared to the lowest (Q1: 8.17 µg/m³ to 9.29 µg/m³) in the fully adjusted model. The second and third quartiles did not show statistically significant results in the fully adjusted model (Q2: HR = 0.99, 95% CI = 0.93 to 1.05, *P* = 0.705, and Q3: HR = 1.01, 95% CI = 0.95 to 1.07, *P* = 0.715). Similarly, our analysis revealed a weaker yet detectable association between PM_2.5 absorbance_ and the incidence of glaucoma, with an HR of 1.03 per IQR increase (95% CI = 1.00 to 1.05, *P* = 0.041) in the maximally adjusted model. However, the associations with larger-sized PM (PM_2.5-10_ and PM_10_) and incident glaucoma were less pronounced and not consistently significant.

**Table 2.** Hazard Ratios for the Association Between Levels of Exposure to PM_2.5_, PM_2.5 absorbance_, PM_2.5-10_, PM_10_ and Glaucoma Incidence

	Univariable Model		Multivariable Model A		Multivariable Model B	
Pollutants	HR (95% CI)	*P* Value	HR (95% CI)	*P* Value	HR (95% CI)	*P* Value
No. of participants/incident cases	448,253/8,999		448,253/8,999		442,754/8,891	
**PM_2.5_**						
Continuous, per IQR increase	1.03 (1.01 to 1.06)	0.008	1.08 (1.06 to 1.11)	<0.001	1.03 (1.00 to 1.06)	0.048
Quartiles						
Q1 (8.17 µg/m³ to 9.29 µg/m³)	Reference		Reference		Reference	
Q2 (9.30 µg/m³ to 9.93 µg/m³)	0.99 (0.94 to 1.05)	0.788	1.02 (0.96 to 1.08)	0.558	0.99 (0.93 to 1.05)	0.705
Q3 (9.94 µg/m³ to 10.56 µg/m³)	1.00 (0.95 to 1.07)	0.885	1.06 (1.00 to 1.13)	0.040	1.01 (0.95 to 1.07)	0.715
Q4 (10.57 µg/m³ to 21.31 µg/m³)	1.10 (1.03 to 1.16)	0.002	1.22 (1.15 to 1.29)	<0.001	1.10 (1.03 to 1.17)	0.005
*P* for trend*		0.002		<0.001		0.004
**PM_2.5 absorbance_**						
Continuous, per IQR increase	1.03 (1.01 to 1.05)	0.010	1.08 (1.06 to 1.1)	<0.001	1.03 (1.00 to 1.05)	0.041
Quartiles						
Q1 (0.83 µg/m³ to 1.00 µg/m³)	Reference		Reference		Reference	
Q2 (1.01 µg/m³ to 1.13 µg/m³)	1.01 (0.95 to 1.07)	0.735	1.02 (0.96 to 1.08)	0.577	1.00 (0.94 to 1.06)	0.919
Q3 (1.14 µg/m³ to 1.30 µg/m³)	1.02 (0.96 to 1.08)	0.500	1.07 (1.01 to 1.14)	0.018	1.02 (0.96 to 1.08)	0.489
Q4 (1.31 µg/m³ to 4.60 µg/m³)	1.06 (1.00 to 1.13)	0.036	1.21 (1.14 to 1.28)	<0.001	1.06 (1.00 to 1.13)	0.062
*P* for trend*		0.037		<0.001		0.046
**PM_2.5-10_**						
Continuous, per IQR increase	1.00 (0.99 to 1.02)	0.654	1.01 (1.00 to 1.03)	0.136	1.00 (0.98 to 1.02)	0.991
Quartiles						
Q1 (5.57 µg/m³ to 5.84 µg/m³)	Reference		Reference		Reference	
Q2 (5.85 µg/m³ to 6.11 µg/m³)	1.03 (0.97 to 1.09)	0.350	1.05 (0.99 to 1.11)	0.117	1.03 (0.97 to 1.09)	0.368
Q3 (6.12 µg/m³ to 6.64 µg/m³)	1.02 (0.96 to 1.08)	0.530	1.07 (1.01 to 1.13)	0.033	1.02 (0.96 to 1.08)	0.590
Q4 (6.65 µg/m³ to 12.82 µg/m³)	1.04 (0.98 to 1.10)	0.227	1.08 (1.02 to 1.15)	0.006	1.02 (0.96 to 1.08)	0.554
P for trend*		0.294		0.006		0.652
**PM_10_**						
Continuous, per SD increase	1.01 (0.99 to 1.03)	0.277	1.03 (1.01 to 1.05)	0.003	1.00 (0.98 to 1.03)	0.741
Quartiles						
Q1 (11.78 µg/m³ to 15.25 µg/m³)	Reference		Reference		Reference	
Q2 (15.26 µg/m³ to 16.03 µg/m³)	1.00 (0.94 to 1.06)	1.000	1.02 (0.96 to 1.08)	0.616	0.99 (0.94 to 1.05)	0.820
Q3 (16.04 µg/m³ to 17.01 µg/m³)	1.01 (0.95 to 1.07)	0.696	1.07 (1.00 to 1.13)	0.034	1.01 (0.95 to 1.07)	0.758
Q4 (17.02 µg/m³ to 31.39 µg/m³)	1.04 (0.98 to 1.10)	0.181	1.11 (1.04 to 1.17)	0.001	1.02 (0.96 to 1.09)	0.481
*P* for trend*		0.164		<0.001		0.406

Asterisk (*) denotes testing for a linear trend. In the multivariable analysis, model A is adjusted for age and sex, whereas model B is adjusted for age, sex, ethnicity, Townsend deprivation index, body mass index, and smoking status. Particulate matter definitions: (PM_2.5_) Finer particles with a diameter less than 2.5 µm; (PM_2.5 absorbance_) Measures light absorption (blackness) of PM_2.5_ filters, served as a proxy of elemental carbon typically emitted from combustion sources; (PM_10_) Particles with a diameter of 10 µm or less; (PM_2.5-10_) Coarse particulate fraction between 2.5 µm and 10 µm in diameter.

CI, confidence interval; HR, hazard ratio; IQR, interquartile range; µg/m³,microgram per cubic meter; PM, particulate matter; Q1, quartile 1; Q2, quartile 2; Q3, quartile 3; Q4, quartiles 4; SD, standard deviation.

Table 3 presents the associations among NO_2_, NO_x_, and the risk of developing glaucoma. In the analysis of NO_x_ exposure, per IQR increase was associated with a marginally significant 2% increased risk (HR = 1.02, 95% CI = 1.00 to 1.04, *P* = 0.067) in the unadjusted model. In the age and sex-adjusted model A, this risk became more pronounced, showing a 6% increase (HR = 1.06, 95% CI = 1.04 to 1.09, *P* < 0.001). However, in the maximally adjusted model B, whereas suggestive of a 2% risk increase, the association did not reach statistical significance (HR = 1.02, 95% CI = 1.00 to 1.04, *P* = 0.123). Quartile-based analysis also showed a similar pattern – the highest quartile of NO_x_ exposure (Q4: 50.78 µg/m³ to 265.94 µg/m³) was associated with a 7% increased risk of incident glaucoma (HR = 1.07, 95% CI = 1.00 to 1.14, *P* = 0.037) compared to the lowest quartile (Q1: 19.74 µg/m³ to 34.38 µg/m³) in the maximally adjusted model. As for NO_2_, the data showed a 3% increase in glaucoma risk (HR = 1.03, 95% CI = 1.00 to 1.05, *P* = 0.045) per IQR increase in the univariable analysis. Furthermore, a 9% increased risk (HR = 1.09, 95% CI = 1.07 to 1.12, *P* < 0.001) was observed in model A adjusted for age and sex. However, this association did not hold in the maximally adjusted model B, neither for per IQR increase nor in the quartile-based analysis.

**Table 3.** Hazard Ratios for the Association Between Levels of Exposure to Nitrogen Oxides and Glaucoma Incidence

	Univariable Model		Multivariable Model A		Multivariable Model B	
Pollutants	HR (95% CI)	*P* Value	HR (95% CI)	*P* Value	HR (95% CI)	*P* Value
No. of participants/incident cases	481,113/9,224		481,113/9,224		475,313/9,113	
**NO_2_**						
Continuous, per IQR increase	1.03 (1.00 to 1.05)	0.045	1.09 (1.07 to 1.12)	<0.001	1.03 (1.00 to 1.06)	0.086
Quartiles						
Q1 (12.93 µg/m³ to 21.47 µg/m³)	Reference		Reference		Reference	
Q2 (21.48 µg/m³ to 26.23 µg/m³)	0.99 (0.93 to 1.05)	0.741	1.01 (0.95 to 1.07)	0.793	0.99 (0.93 to 1.05)	0.687
Q3 (26.24 µg/m³ to 31.25 µg/m³)	1.02 (0.96 to 1.08)	0.524	1.07 (1.01 to 1.13)	0.023	1.02 (0.96 to 1.08)	0.600
Q4 (31.26 µg/m³ to 108.49 µg/m³)	1.04 (0.98 to 1.10)	0.219	1.19 (1.12 to 1.26)	<0.001	1.04 (0.98 to 1.11)	0.210
*P* for trend*		0.141		<0.001		0.148
**NO_x_**						
Continuous, per IQR increase	1.02 (1.00 to 1.04)	0.067	1.06 (1.04 to 1.09)	<0.001	1.02 (1.00 to 1.04)	0.123
Quartiles						
Q1 (19.74 µg/m³ to 34.38 µg/m³)	Reference		Reference		Reference	
Q2 (34.39 µg/m³ to 42.41 µg/m³)	1.02 (0.96 to 1.08)	0.487	1.04 (0.99 to 1.11)	0.145	1.02 (0.96 to 1.09)	0.445
Q3 (42.42 µg/m³ to 50.77 µg/m³)	1.03 (0.97 to 1.09)	0.401	1.09 (1.03 to 1.15)	0.005	1.04 (0.98 to 1.10)	0.253
Q4 (50.78 µg/m³ to 265.94 µg/m³)	1.06 (1.00 to 1.13)	0.039	1.20 (1.13 to 1.27)	<0.001	1.07 (1.00 to 1.14)	0.037
*P* for trend*		0.045		<0.001		0.037

Asterisk (*) denotes testing for a linear trend. In the multivariable analysis, model A is adjusted for age and sex, whereas model B is adjusted for age, sex, ethnicity, Townsend deprivation index, body mass index, and smoking status.

CI, confidence interval; HR, hazard ratio; IQR, interquartile range; µg/m³, microgram per cubic meter; NO_2_, nitrogen dioxide; NO_x_, nitrogen oxides; Q1, quartile 1; Q2, quartile 2; Q3, quartile 3; Q4, quartiles 4.

**Figure 2.**
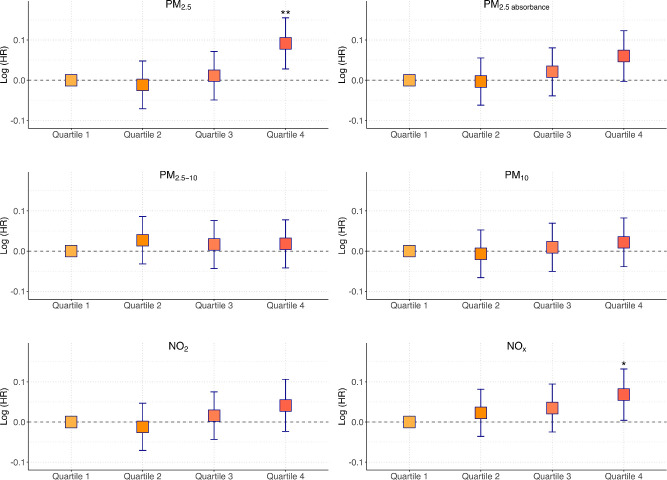
Visual Representation of adjusted HRs across quartiles of air pollution exposure. HRs have been derived from a maximally adjusted model (refer to Table 2 and Table 3, model B) that incorporates age, sex, ethnicity, Townsend deprivation index, body mass index, and smoking status as covariates. On the Y-axis, the HRs are presented on a log scale via a natural logarithm transformation (base *e*). A *horizontal dashed gray line* denotes the no-effect threshold, corresponding to an HR of 1. Error bars indicate 95% confidence intervals. Asterisks highlight statistically significant differences, with * indicating *P* < 0.05, and ** indicating *P* < 0.01. PM, particulate matter; NO_2_, nitrogen dioxide; NO_x_, nitrogen oxides; HR, hazard ratio. Definitions for particulate matter include: PM_2.5_, finer particles with a diameter of less than 2.5 µm; PM_2.5 absorbance_, a measure of the light absorption (blackness) of PM_2.5_ filters, serving as a proxy for elemental carbon typically emitted from combustion sources; PM_2.5-10_, coarse particulate fraction between 2.5 µm and 10 µm in diameter; PM_10_, particles with a diameter of 10 µm or less.

Figure 2 shows the adjusted HRs across quartiles of air pollution exposure, as estimated from the maximally adjusted model (model B).

In the sensitivity analysis, we further excluded participants who had not resided at the address recorded during recruitment before 2005, which constituted approximately 14% of the entire cohort. This left us with non-movers who had been at their residential address for more than 5 years prior to the study baseline (January 1, 2010) for analysis. We observed consistent results with those for the full cohort (Supplementary Table S1).

In another sensitivity analysis, we additionally adjusted for the Charlson comorbidity index to account for potential ascertainment bias related to comorbidities and hospitalizations.^26^ As shown in Supplementary Table S2, we observed a slight attenuation of the associations; however, the overall findings remained consistent with the primary analysis.

In the gene-environment interaction analyses (Supplementary Table S3), we did not observe any statistically significant associations (all *P* interactions > 0.05), suggesting that genetic predisposition to glaucoma, as quantified by the multitrait PRS, does not modify the effect of air pollution exposure on glaucoma risk.

## Discussion

To the best of our knowledge, this is the largest longitudinal study to examine the association between air pollution and the risk of developing glaucoma. In this large cohort of 481,113 participants, we observed a robust association between PM_2.5_ exposure and the incidence of glaucoma. Additionally, whereas the associations with PM_2.5absorbance_, NO_x_, and NO_2_ were weaker than that with PM_2.5_, they remained marginally statistically significant, hinting at a potential link. In contrast, exposure to PM_2.5-10_, and PM_10_ did not demonstrate statistically significant associations after controlling for potential confounders.

The landmark Harvard Six Cities Study provided concrete evidence of the association between mortality and air pollution, particularly PM_2.5_.^27^^–^^29^ In concordance, our study found PM_2.5_ to be the air pollutant most strongly associated with the risk of glaucoma, which is in line with current literature. Utilizing data from the UK Biobank, Chua et al.^14^ reported a 6% increase in the odds of glaucoma per IQR (1.07 µg/m^3^) increase in PM_2.5_. Similarly, Grant and colleagues observed a 14% increase in the odds of glaucoma per IQR (2.5 µg/m^3^) increase in PM_2.5_ levels in the Canadian Longitudinal Study on Aging (CLSA) cohort.^15^ In a rural China cohort, Yang et al. observed a 7% and a 14% increase in the odds of glaucoma and primary angle-closure glaucoma (PACG), respectively, for every 10 µg/m^3^ rise in PM_2.5_ concentration.^17^ Additionally, a study conducted on the Taiwanese population revealed a 19% greater likelihood of primary open-angle glaucoma (POAG) diagnosis for each 25 µg/m^3^ increment in PM_2.5_ level.^16^ Despite varying PM_2.5_ levels across geographic locations—illustrated by the CLSA cohort's average PM_2.5_ concentration being substantially lower (mean = 6.5 µg/m^3^) than the lower bound observed in our study (range = 8.17–21.32 µg/m^3^, median = 9.93 µg/m^3^), and the median level in rural China (median = 59.8 µg/m^3^, range = 28.0–96.4 µg/m^3^) as reported by Yang et al. being nearly sixfold higher than ours—these data point toward a globally consistent correlation between PM_2.5_ and glaucoma risk. Furthermore, a recent longitudinal study that investigated glaucoma-related traits suggested that a higher level of PM_2.5_ is associated with a faster rate of retinal nerve fiber layer (RNFL) thinning.^30^ Another recent investigation into acute primary angle closure – the “precursor” to PACG – also supports the association between higher PM_2.5_ level and increased odds of this condition.^31^ Taken together, existing evidence supports the detrimental effect of PM_2.5_ on the development of glaucoma.

PM_2.5 absorbance_ measures the light absorbed by PM_2.5_ filters, serving as a proxy for elemental carbon emitted from combustion sources, such as vehicle exhaust. In this study, we observed a marginally significant association between PM_2.5 absorbance_ and the risk of glaucoma. This observation is consistent with our prior cross-sectional analysis, which demonstrated that higher PM_2.5 absorbance_ level is associated with thinner RNFL, further suggesting a potential link between PM_2.5 absorbance_ and glaucoma.^32^ One plausible underlying pathophysiological mechanism could be oxidative stress. Supporting this hypothesis, existing research has found that long-term exposure to ambient black carbon is associated with increased IOP, particularly in individuals with a genetic predisposition to oxidative stress.^33^ Moreover, black carbon is an ideal and potent carrier of various toxic substances that adhere to its surface.^34^ When these combined particles are deposited onto ocular tissues or enter the bloodstream, the toxic load could be amplified. This effect can be particularly pronounced in regions with high levels of air pollution; therefore, although the statistical significance of our results is marginal, the potential global health implications should not be overlooked. Given that the range of PM_2.5 absorbance_ in our study was relatively narrow, this might have contributed to the marginal significance observed. Future research in diverse geographic settings with wider exposure variations is essential to confirm our findings.

Research has increasingly indicated that chronic exposure to NO_2_ and NO_x_ is linked to various adverse health outcomes, including respiratory conditions, cardiovascular diseases, and now, as our study suggests, the incidence of glaucoma.^35^^–^^38^ In brief, NO_2_ and NO_x_ are gaseous pollutants primarily resulting from traffic-related emissions and other combustion processes. NO_x_ is a broader term which includes several nitrogen oxides, such as NO and NO_2_. Our data demonstrated modest and marginally significant associations among NO_2_, NO_x_, and the risk of incident glaucoma. However, caution is warranted in interpreting these findings due to the strong correlation between NO_x_ (which includes NO_2_) and PM_2.5_, more so than with other particulates (*r* = 0.87; Supplementary Table S4). The relationship among NO_x_, NO_2_, and PM_2.5_ is complex, involving both direct and indirect interactions, which we think stems from their shared emission sources, the role of NO_2_ in forming secondary PM_2.5_, and regulatory measures that tend to reduce both NO_x_ and PM_2.5_ concurrently, and vice versa. Therefore, in this study, it is challenging to distinguish the effect of NO_x_ from those of PM_2.5_, and more research is needed to further explore the exact roles of NO_x_ and NO_2_ on glaucoma pathogenesis.

The estimation of ambient air pollution levels in our study was based solely on participants’ home addresses, which inherently omits indoor pollutant exposure as well as exposure during commuting or in workplace settings. According to the Environmental Protection Agency, the levels of air pollution may be two to five times higher indoors than outdoors.^39^ Studies have also shown that the average personal exposure levels in road transport modes are typically double those recorded at urban background fixed site monitors. Moreover, the mode of travel matters; exposure levels on the London Underground can be three to eight times greater compared to surface transport.^40^^,^^41^ Therefore, it becomes evident that our primary exposure measures are imperfect, capturing only a fraction—perhaps merely the “tip of the iceberg”—of an individual's true exposure to air pollutants.

Strengths of our study include its prospective, longitudinal design with a substantial follow-up period (nearly 13 years), and a very large sample size, which provides unprecedented statistical power to examine the link between ambient air pollution and glaucoma incidence. However, we acknowledge several limitations:

(1) Selection bias might exist because the UK Biobank is not representative of the sampling population. Nonetheless, valid assessment of exposure-disease relationships may be widely generalizable and does not require participants to be representative of the population at large.^42^
(2) We cannot account for cumulative lifetime exposures or non-residential sources of exposures to air pollution. Even within the scope of residential sources, exposure misclassification in longitudinal studies can occur due to residential mobility. In this study, ambient air pollution levels were estimated for the year of 2010, which we hope reflects long-term exposures. However, it is possible that some participants may have moved around before 2010, or between 2010 and the time of their glaucoma diagnosis. We conducted a sensitivity analysis for non-movers before 2010 (see Supplementary Table S1). Consistent results were observed when restricting to those who did not change residential addresses for more than 5 years prior to the study baseline (January 1, 2010). Our results, along with a previous report suggesting that individuals often move to areas with similar air pollution exposure levels, imply that the potential bias due to residential mobility was small.^43^
(3) Case ascertainment was derived from hospital inpatient admission records, which did not include outpatient data. It is noteworthy that a considerable proportion of patients with glaucoma receive care in outpatient clinics, especially in early stages of the disease. On occasions where patients are managed exclusively in outpatient settings, their glaucoma diagnosis would be documented only upon subsequent hospitalization for other diseases, for example, strokes or heart attacks. In addition, if a patient, who is otherwise healthy, never undergoes hospital admission, their case would not be captured at all. This method of case ascertainment could potentially result in an overestimation of the latency period before glaucoma detection and an underestimation of its actual incidence. Moreover, it may introduce bias, considering the well-documented relationship between air pollution and general health. Furthermore, there is an additional consideration that individuals with higher socioeconomic status may receive care through private healthcare systems, which are not recorded in NHS ICD billing codes.
(4) Our outcome measure was restricted to all-cause glaucoma without differentiating among its major subtypes — such as POAG, PACG, and pseudoexfoliative glaucoma — due to potential inaccuracies in the NHS ICD coding system for classifying glaucoma subtypes.

Our study provides strong evidence linking ambient air pollution to an increased risk of developing glaucoma within the UK Biobank cohort. However, the generalizability of these findings to other regions must be considered with caution. The levels and composition of air pollutants, as well as demographic, environmental, and healthcare factors, can vary significantly across different geographic locations. Notably, PM_2.5_ concentrations in our cohort ranged from 8.17 to 21.32 µg/m³, which is substantially below the global average of 30.6 µg/m³ estimated in 2010.^44^ This relatively narrow range of exposure may limit the generalizability of our findings globally, especially to regions with higher levels of air pollution. Additionally, in the UK Biobank cohort, PM_2.5_ and NO_2_ levels among all participants exceeded the annual maximum level suggested by the latest WHO guidelines, thereby preventing an assessment of associations at or below these thresholds. Furthermore, healthcare infrastructure and access to glaucoma screening may differ globally, influencing both the detection and management of glaucoma. Therefore, although the association between air pollution and glaucoma appears consistent across various studies, including those conducted in Canada,^15^ mainland China,^17^ and Taiwan,^16^ the magnitude and specific dose-response relationships may vary. Further research involving diverse populations and regions is needed to validate our findings and assess their broader applicability in various environmental contexts.

In summary, our study has established a clear correlation between higher levels of ambient air pollutants, particularly PM_2.5_, and an increased risk of glaucoma development. These results underscore the potential public health impact of ambient air pollution on glaucoma risk and highlight the urgent need for further research into targeted environmental interventions in this domain.

## Supplementary Material

Supplement 1

Supplement 2

Supplement 3

Supplement 4

Supplement 5

## References

[1] Taylor L. WHO cuts air pollution limits to save millions of lives. *BMJ*. 2021; 374: n2349. 34556465 10.1136/bmj.n2349

[2] Papadogeorgou G, Kioumourtzoglou M-A, Braun D, Zanobetti A. Low levels of air pollution and health: effect estimates, methodological challenges, and future directions. *Curr Environ Health Rep*. 2019; 6(3): 105–115. 31090042 10.1007/s40572-019-00235-7 PMC7161422

[3] Stafoggia M, Oftedal B, Chen J, et al. Long-term exposure to low ambient air pollution concentrations and mortality among 28 million people: results from seven large European cohorts within the ELAPSE project. *Lancet Planet Health*. 2022; 6(1): e9–e18. 34998464 10.1016/S2542-5196(21)00277-1

[4] Vedal S, Brauer M, White R, Petkau J. Air pollution and daily mortality in a city with low levels of pollution. *Environ Health Perspect*. 2003; 111(1): 45–52. 12515678 10.1289/ehp.5276 PMC1241305

[5] World Health Organization. *WHO global air quality guidelines: particulate matter (PM2.5 and PM10), ozone, nitrogen dioxide, sulfur dioxide and carbon monoxide*. World Health Organization; 2021. Available at: https://www.who.int/publications/i/item/9789240034228#:∼:text=In%202015,%20the%20World%20Health%20Assembly. 34662007

[6] World Health Organization. *Air quality guidelines: global update 2005: particulate matter, ozone, nitrogen dioxide, and sulfur dioxide*. World Health Organization; 2006. Available at: https://www.who.int/publications/i/item/WHO-SDE-PHE-OEH-06.02#:∼:text=Knowledge%20about%20the%20hazardous%20properties. 34662007

[7] GBD 2019 Blindness and Vision Impairment Collaborators; Vision Loss Expert Group of the Global Burden of Disease Study. Causes of blindness and vision impairment in 2020 and trends over 30 years, and prevalence of avoidable blindness in relation to VISION 2020: the Right to Sight: an analysis for the global burden of disease study. *Lancet Glob Health*. 2021; 9(2): e144–e160. 33275949 10.1016/S2214-109X(20)30489-7 PMC7820391

[8] Vijaya L, George R, Baskaran M, et al. Prevalence of primary open-angle glaucoma in an urban south Indian population and comparison with a rural population: the Chennai glaucoma study. *Ophthalmology*. 2008; 115(4): 648–654. e1. 17664010 10.1016/j.ophtha.2007.04.062

[9] Risom L, Møller P, Loft S. Oxidative stress-induced DNA damage by particulate air pollution. *Mutat Res*. 2005; 592(1-2): 119–137. 16085126 10.1016/j.mrfmmm.2005.06.012

[10] Pope CA. 3rd Epidemiology of fine particulate air pollution and human health: biologic mechanisms and who's at risk? *Environ Health Perspect*. 2000; 108(Suppl 4): 713–723. 10931790 10.1289/ehp.108-1637679 PMC1637679

[11] Lin CC, Chiu CC, Lee PY, et al. The adverse effects of air pollution on the eye: a review. *Int J Environ Res Public Health*. 2022; 19(3): 1186. 35162209 10.3390/ijerph19031186 PMC8834466

[12] Trouilloud A, Ferry E, Boucart M, et al. Impact of glaucoma on the spatial frequency processing of scenes in central vision. *Vis Neurosci*. 2023; 40: E001. 36752177 10.1017/S0952523822000086 PMC9970733

[13] Grant A, Leung G, Freeman EE. Ambient air pollution and age-related eye disease: a systematic review and meta-analysis. *Invest Ophthalmol Vis Sci*. 2022; 63(9): 17. 10.1167/iovs.63.9.17 PMC9396677 35960515

[14] Chua SY, Khawaja AP, Morgan J, et al. The relationship between ambient atmospheric fine particulate matter (PM2.5) and glaucoma in a large community cohort. *Invest Ophthalmol Vis Sci*. 2019; 60(14): 4915–4923. 31764948 10.1167/iovs.19-28346

[15] Grant A, Leung G, Aubin M-J, Kergoat M-J, Li G, Freeman EE. Fine particulate matter and age-related eye disease: the Canadian longitudinal study on aging. *Invest Ophthalmol Vis Sci*. 2021; 62(10): 7. 10.1167/iovs.62.10.7 PMC8354031 34369984

[16] Sun H-Y, Luo C-W, Chiang Y-W, et al. Association between PM2.5 exposure level and primary open-angle glaucoma in Taiwanese adults: a nested case–control study. *Int J Environ Res Public Health*. 2021; 18(4): 1714. 33578928 10.3390/ijerph18041714 PMC7916685

[17] Yang X, Yang Z, Liu Y, et al. The association between long-term exposure to ambient fine particulate matter and glaucoma: a nation-wide epidemiological study among Chinese adults. *Int J Hyg Environ Health*. 2021; 238: 113858. 34634756 10.1016/j.ijheh.2021.113858 PMC9190215

[18] Li L, Zhu Y, Han B, et al. Acute exposure to air pollutants increase the risk of acute glaucoma. *BMC Public Health*. 2022; 22(1): 1782. 36127653 10.1186/s12889-022-14078-9 PMC9487138

[19] Min K-B, Min J-Y. Association of ambient particulate matter exposure with the incidence of glaucoma in childhood. *Am J Ophthalmol*. 2020; 211: 176–182. 31734134 10.1016/j.ajo.2019.11.013

[20] Craig JE, Han X, Qassim A, et al. Multitrait analysis of glaucoma identifies new risk loci and enables polygenic prediction of disease susceptibility and progression. *Nat Genet*. 2020; 52(2): 160–166. 31959993 10.1038/s41588-019-0556-y PMC8056672

[21] Beelen R, Hoek G, Vienneau D, et al. Development of NO2 and NOx land use regression models for estimating air pollution exposure in 36 study areas in Europe – the ESCAPE project. *Atmos Environ*. 2013; 72: 10–23.

[22] Eeftens M, Tsai M-Y, Ampe C, et al. Spatial variation of PM2.5, PM10, PM2.5 absorbance and PMcoarse concentrations between and within 20 European study areas and the relationship with NO2–results of the ESCAPE project. *Atmos Environ*. 2012; 62: 303–317.

[23] Feng S, Gao D, Liao F, Zhou F, Wang X. The health effects of ambient PM2.5 and potential mechanisms. *Ecotoxicol Environ Saf*. 2016; 128: 67–74. 26896893 10.1016/j.ecoenv.2016.01.030

[24] Miller FJ, Gardner DE, Graham JA, Lee RE Jr, Wilson WE, Bachmann JD. Size considerations for establishing a standard for inhalable particles. *J Air Pollut Control Assoc*. 1979; 29(6): 610–615.

[25] Pinkerton KE, Green F, Saiki C, et al. Distribution of particulate matter and tissue remodeling in the human lung. *Environ Health Perspect*. 2000; 108(11): 1063–1069. 11102298 10.1289/ehp.001081063 PMC1240164

[26] Charlson ME, Pompei P, Ales KL, MacKenzie CR. A new method of classifying prognostic comorbidity in longitudinal studies: development and validation. *J Chronic Dis*. 1987; 40(5): 373–383. 3558716 10.1016/0021-9681(87)90171-8

[27] Dockery DW, Pope CA, Xu X, et al. An association between air pollution and mortality in six US cities. *N Engl J Med*. 1993; 329(24): 1753–1759. 8179653 10.1056/NEJM199312093292401

[28] Lepeule J, Laden F, Dockery D, Schwartz J. Chronic exposure to fine particles and mortality: an extended follow-up of the Harvard Six Cities study from 1974 to 2009. *Environ Health Perspect*. 2012; 120(7): 965–970. 22456598 10.1289/ehp.1104660 PMC3404667

[29] Laden F, Schwartz J, Speizer FE, Dockery DW. Reduction in fine particulate air pollution and mortality: extended follow-up of the Harvard Six Cities study. *Am J Respir Crit Care Med*. 2006; 173(6): 667–672. 16424447 10.1164/rccm.200503-443OC PMC2662950

[30] Gayraud L, Mortamais M, Schweitzer C, et al. Association of long-term exposure to ambient air pollution with retinal neurodegeneration: the prospective Alienor study. *Environ Res*. 2023; 232: 116364. 37301495 10.1016/j.envres.2023.116364

[31] Wu N, Shi W, Sun X. Association of Long-term exposure to ambient air pollution with the risk of acute primary angle closure. *Transl Vis Sci Technol*. 2024; 13(3): 7. 10.1167/tvst.13.3.7 PMC10941992 38470319

[32] Chua SY, Khawaja AP, Dick AD, et al. Ambient air pollution associations with retinal morphology in the UK Biobank. *Invest Ophthalmol Vis Sci*. 2020; 61(5): 32. 10.1167/iovs.61.5.32 PMC7405693 32428233

[33] Nwanaji-Enwerem JC, Wang W, Nwanaji-Enwerem O, et al. Association of long-term ambient black carbon exposure and oxidative stress allelic variants with intraocular pressure in older men. *JAMA Ophthalmol*. 2019; 137(2): 129–137. 30419128 10.1001/jamaophthalmol.2018.5313 PMC6440251

[34] Janssen NA, Gerlofs-Nijland ME, Lanki T, et al. *Health effects of black carbon*. World Health Organization. Regional Office for Europe; 2012. Available at: https://www.who.int/europe/publications/i/item/9789289002653#:∼:text=This%20report%20presents%20the%20results%20of%20a.

[35] Huang Y, Zhu M, Ji M, et al. Air pollution, genetic factors, and the risk of lung cancer: a prospective study in the UK Biobank. *Am J Respir Crit Care Med*. 2021; 204(7): 817–825. 34252012 10.1164/rccm.202011-4063OC

[36] Hamra GB, Laden F, Cohen AJ, Raaschou-Nielsen O, Brauer M, Loomis D. Lung cancer and exposure to nitrogen dioxide and traffic: a systematic review and meta-analysis. *Environ Health Perspect*. 2015; 123(11): 1107–1112. 25870974 10.1289/ehp.1408882 PMC4629738

[37] de Bont J, Jaganathan S, Dahlquist M, Persson Å, Stafoggia M, Ljungman P. Ambient air pollution and cardiovascular diseases: an umbrella review of systematic reviews and meta-analyses. *J Intern Med*. 2022; 291(6): 779–800. 35138681 10.1111/joim.13467 PMC9310863

[38] Faustini A, Rapp R, Forastiere F. Nitrogen dioxide and mortality: review and meta-analysis of long-term studies. *Eur Respir J*. 2014; 44(3): 744–753. 24558178 10.1183/09031936.00114713

[39] Environmental Protection Agency. Indoor Air Quality (IAQ). Volatile organic compounds’ impact on indoor air quality. Updated August 13, 2024. Available at: https://www.epa.gov/indoor-air-quality-iaq/volatile-organic-compounds-impact-indoor-air-quality.

[40] Adams HS, Nieuwenhuijsen MJ, Colvile RN, McMullen MA, Khandelwal P. Fine particle (PM2.5) personal exposure levels in transport microenvironments, London, UK. *Sci Total Environ*. 2001; 279(1-3): 29–44. 11712603 10.1016/s0048-9697(01)00723-9

[41] Smith J, Barratt B, Fuller G, et al. PM2.5 on the London Underground. *Environ Int*. 2020; 134: 105188. 31787325 10.1016/j.envint.2019.105188 PMC6902242

[42] Fry A, Littlejohns TJ, Sudlow C, et al. Comparison of sociodemographic and health-related characteristics of UK biobank participants with those of the general population. *Am J Epidemiol*. 2017; 186(9): 1026–1034. 28641372 10.1093/aje/kwx246 PMC5860371

[43] Oudin A, Forsberg B, Strömgren M, Beelen R, Modig L. Impact of residential mobility on exposure assessment in longitudinal air pollution studies: a sensitivity analysis within the ESCAPE project. *Scientific World Journal*. 2012; 2012(1): 125818. 23251098 10.1100/2012/125818 PMC3515908

[44] Statista. Annual average particulate pollution (PM2.5) levels worldwide from 2000 to 2021. Accessed August 15, 2024, Available at: https://www.statista.com/statistics/1464237/global-annual-average-pm25-concentrations/.

