# Supplementary material for: Association of Ambient Air Pollution Exposure With Incident Glaucoma: 12-Year Evidence From the UK Biobank Cohort
Source: Invest Ophthalmol Vis Sci. 2024 Oct 16;65(12):22. doi: 10.1167/iovs.65.12.22 (PMC11488522; doi:10.1167/iovs.65.12.22)
Supplement: Supplement 1 [file iovs-65-12-22_s001.pdf]

ASSESSMENT OF COVARIABLES

1. Age: Age was determined by subtracting the date of birth from January 1, 2010. The result is expressed in years (refer to data fields 33 and 52).

2. Sex: Sex data was acquired from the central registry upon recruitment and may also include subsequent updates by participants. Therefore, this field can reflect both NHS-recorded and self-reported sex (data field 31).

3. Ethnicity: The response options for ethnicity included: White (English/Irish or other White backgrounds), Asian or British Asian (Indian/Pakistani/Bangladeshi or other Asian backgrounds), Black or Black British (Caribbean, African, or other Black backgrounds), Chinese, Mixed (White and Black Caribbean or African, White and Asian, or other Mixed backgrounds), and Other ethnic groups (not defined). For analysis purposes, ethnicity was categorized into two groups: White and Others.

4. Townsend Deprivation Index: This index was derived from the participant's postcode at recruitment and the corresponding output area from the preceding national census. The index calculation incorporates factors such as employment status, home ownership, car ownership, and household conditions. A higher, more positive index value indicates greater social deprivation (data-field 22189).

5. Body Mass Index: Weight and height were collected when participants attended the initial assessment centre from 2006 to 2010. Height was measured in centimeters using a Seca 202 device, and weight was measured in kilograms using various methods. These measurements were amalgamated into a single weight variable in the UK Biobank release data. Body mass index was calculated as weight divided by the square of height (kg/m^2).

6. Smoking Status: Smoking status was determined by the response to the question "Do you smoke tobacco now?" The options provided were "Current," "Previous," "Never," and "Prefer not to answer."

7. Charlson Comorbidity Index (CCI): The CCI quantifies the comorbidity burden of participants by assigning weighted scores to various conditions as follows:

- <u>Myocardial Infarction</u> (ICD-10 codes: I21, I22, I252) – 1 point
- <u>Congestive Heart Failure</u> (ICD-10 codes: I50, I110, I130, I132) – 1 point
- <u>Peripheral Vascular Disease</u> (ICD-10 codes: I70, I71, I731, I738, I739, I771, K551, K558, K559, Z958, Z959) – 1 point
- <u>Cerebrovascular Disease</u> (ICD-10 codes: I60, I61, I62, I63, I64, G45, G46, I678) – 1 point
- <u>Dementia</u> (ICD-10 codes: F00, F01, F02, F03, G30) – 1 point
- <u>Chronic Pulmonary Disease</u> (ICD-10 codes: J40, J41, J42, J43, J44, J45, J46, J47, J60, J61, J62, J63, J64, J65, J66, J67, J684, J701, J703) – 1 point

- Rheumatic Disease (ICD-10 codes: M05, M06, M31, M32, M33, M34, M35, M36) – 1 point
- Peptic Ulcer Disease (ICD-10 codes: K25, K26, K27, K28) – 1 point
- Mild Liver Disease (ICD-10 codes: B18, K70, K71, K73, K74, K760, K762, K763, K764) – 1 point
- Diabetes without Complications (ICD-10 codes: E100, E101, E106, E108, E109, E110, E111, E116, E118, E119) – 1 point
- Diabetes with Complications (ICD-10 codes: E102, E103, E104, E105, E107, E112, E113, E114, E115, E117) – 2 points
- Hemiplegia or Paraplegia (ICD-10 codes: G81, G82, G041, G114, G801, G802) – 2 points
- Renal Disease (ICD-10 codes: I12, I13, N03, N05, N18, N19, N25, Z490, Z491, Z492, Z940, Z992) – 2 points
- Cancer (ICD-10 codes: C00, C01, C02, C03, C04, C05, C06, C07, C08, C09, C10, C11, C12, C13, C14, C15, C16, C17, C18, C19, C20, C21, C22, C23, C24) – 2 points
- Metastatic Solid Tumour (ICD-10 codes: C77, C78, C79, C80) – 6 points
- AIDS/HIV (ICD-10 codes: B20, B21, B22, B24) – 6 points

The computation of the CCI was facilitated using the 'comorbidity' package in R.

GENOTYPING AND POLYGENIC RISK SCORE (PRS)

Genetic data for approximately 490,000 participants were generated using two closely related genotyping platforms. The Affymetrix UK BiLEVE Axiom Array returned genotypes at 807,411 markers for approximately 50,000 participants, whereas the Affymetrix UK Biobank Axiom Array provided genotypes at 825,925 markers for the remaining approximately 440,000 participants.[1] Quality control and imputation, a genotypic determination based on inference and not by direct typing, were performed jointly for these two platforms, as previously described.[2] The reference panels used for imputation were the UK10K and the Haplotype Reference Consortium.

To assess whether observed exposure-outcome associations were modified by genetic factors (gene-environment interaction), we constructed a polygenic risk score (PRS) based on 2,673 independent single nucleotide polymorphisms (SNPs) associated with glaucoma (at $P \leq 0.001$) from a recent multitrait analysis of genome-wide association studies (MTAG), which included UK Biobank data.[3] Glaucoma is a complex polygenic disease, and we considered the MTAG PRS to be a more accurate representation of genetic variation in glaucoma than any individual or limited set of variants. We used the effect estimates from the original MTAG study to generate a glaucoma PRS for each participant using a standard weighted sum of individual SNPs:

$$\sum_{i=1}^{2673} \beta(i) \times SNP(i)$$

where $\beta(i)$ is the estimated effect size of $SNP(i)$ on glaucoma. This glaucoma MTAG PRS has been demonstrated to predict earlier age at glaucoma diagnosis, progression of the disease, and the need for surgical intervention in an independent cohort.[3]

REFERENCES

1. Wain LV, Shrine N, Miller S, et al. Novel insights into the genetics of smoking behaviour, lung function, and chronic obstructive pulmonary disease (UK BiLEVE): a genetic association study in UK Biobank. *The Lancet Respiratory Medicine*. 2015;3(10):769-781.
2. Bycroft C, Freeman C, Petkova D, et al. The UK Biobank resource with deep phenotyping and genomic data. *Nature*. 2018;562(7726):203-209.
3. Craig JE, Han X, Qassim A, et al. Multitrait analysis of glaucoma identifies new risk loci and enables polygenic prediction of disease susceptibility and progression. *Nature genetics*. 2020;52(2):160-166.
