# Supplementary material for: Association of Ambient Air Pollution Exposure With Incident Glaucoma: 12-Year Evidence From the UK Biobank Cohort
Source: Invest Ophthalmol Vis Sci. 2024 Oct 16;65(12):22. doi: 10.1167/iovs.65.12.22 (PMC11488522; doi:10.1167/iovs.65.12.22)
Supplement: Supplement 2 [file iovs-65-12-22_s002.pdf]

Table S1. Sensitivity analysis of non-movers.

Pollutants	Univariable Model		Multivariable Model A		Multivariable Model B	
	HR (95% CI)	*P*	HR (95% CI)	*P*	HR (95% CI)	*P*
No. of participants / incident cases	384138 / 7963		384138 / 7963		379889 / 7868	
**$PM_{2.5}$**						
Continuous, per IQR increase	1.04 (1.01 to 1.07)	0.003	1.09 (1.06 to 1.11)	<0.001	1.03 (1.00 to 1.06)	0.045
Quartiles						
Quartile 1	Reference		Reference		Reference	
Quartile 2	0.99 (0.93 to 1.05)	0.654	1.01 (0.95 to 1.08)	0.713	0.98 (0.92 to 1.05)	0.615
Quartile 3	0.99 (0.93 to 1.06)	0.791	1.05 (0.98 to 1.12)	0.155	1.00 (0.94 to 1.06)	0.944
Quartile 4	1.10 (1.04 to 1.17)	0.002	1.22 (1.14 to 1.29)	<0.001	1.09 (1.02 to 1.17)	0.009
P for trend*		0.002		<0.001		0.010
**$PM_{2.5\ absorbance}$**						
Continuous, per IQR increase	1.04 (1.02 to 1.07)	0.001	1.08 (1.06 to 1.1)	<0.001	1.03 (1.00 to 1.06)	0.039
Quartiles						
Quartile 1	Reference		Reference		Reference	
Quartile 2	1.02 (0.96 to 1.08)	0.587	1.02 (0.96 to 1.09)	0.473	1.00 (0.94 to 1.07)	0.928
Quartile 3	1.04 (0.97 to 1.10)	0.272	1.08 (1.02 to 1.15)	0.015	1.03 (0.97 to 1.10)	0.379
Quartile 4	1.10 (1.03 to 1.16)	0.004	1.21 (1.14 to 1.29)	<0.001	1.07 (1.00 to 1.14)	0.057
P for trend*		0.003		<0.001		0.043

**$PM_{2.5-10}$**						
Continuous, per IQR increase	1.01 (0.99 to 1.03)	0.317	1.01 (1.00 to 1.03)	0.136	1.00 (0.98 to 1.02)	0.991
Quartiles						
Quartile 1	Reference		Reference		Reference	
Quartile 2	1.02 (0.96 to 1.08)	0.596	1.03 (0.97 to 1.10)	0.337	1.01 (0.95 to 1.08)	0.777
Quartile 3	1.03 (0.97 to 1.09)	0.386	1.06 (1.00 to 1.13)	0.050	1.01 (0.95 to 1.08)	0.672
Quartile 4	1.05 (0.99 to 1.12)	0.110	1.09 (1.02 to 1.16)	0.007	1.02 (0.96 to 1.09)	0.500
P for trend*		0.105		0.004		0.496
**PM_{10}**						
Continuous, per SD increase	1.02 (1.00 to 1.04)	0.119	1.03 (1.01 to 1.06)	0.002	1.01 (0.98 to 1.03)	0.563
Quartiles						
Quartile 1	Reference		Reference		Reference	
Quartile 2	0.99 (0.93 to 1.06)	0.776	1.01 (0.95 to 1.07)	0.848	0.98 (0.92 to 1.05)	0.615
Quartile 3	1.03 (0.96 to 1.09)	0.410	1.07 (1.01 to 1.14)	0.027	1.02 (0.95 to 1.08)	0.613
Quartile 4	1.05 (0.99 to 1.12)	0.093	1.11 (1.04 to 1.18)	0.001	1.02 (0.96 to 1.09)	0.461
P for trend*		0.052		<0.001		0.313
No. of participants / incident cases	413609 / 8175		413609 / 8175		409126 / 8077	
**NO_2**						
Continuous, per IQR increase	1.04 (1.01 to 1.07)	0.012	1.09 (1.06 to 1.12)	<0.001	1.03 (0.99 to 1.06)	0.108
Quartiles						

Quartile 1	Reference		Reference		Reference	
Quartile 2	0.99 (0.96 to 1.05)	0.641	1.00 (0.94 to 1.07)	0.919	0.99 (0.93 to 1.05)	0.637
Quartile 3	1.02 (0.96 to 1.09)	0.509	1.07 (1.00 to 1.13)	0.044	1.02 (0.95 to 1.08)	0.641
Quartile 4	1.05 (0.99 to 1.12)	0.108	1.18 (1.11 to 1.26)	<0.001	1.04 (0.97 to 1.11)	0.279
P for trend*		0.060		<0.001		0.197
**NO_x**						
Continuous, per IQR increase	1.03 (1.00 to 1.05)	0.026	1.06 (1.04 to 1.09)	<0.001	1.02 (0.99 to 1.05)	0.141
Quartiles						
Quartile 1	Reference		Reference		Reference	
Quartile 2	1.02 (0.96 to 1.09)	0.500	1.04 (0.98 to 1.11)	0.171	1.03 (0.96 to 1.09)	0.433
Quartile 3	1.01 (0.95 to 1.07)	0.855	1.06 (1.00 to 1.13)	0.063	1.01 (0.95 to 1.08)	0.691
Quartile 4	1.08 (1.01 to 1.15)	0.017	1.20 (1.13 to 1.27)	<0.001	1.07 (1.00 to 1.15)	0.039
P for trend*		0.036		<0.001		0.067

In this sensitivity analysis, we further excluded participants who had not resided at the address recorded during recruitment before 2005, this left us with non-movers who had been at their residential address for more than 5 years prior to the study baseline. Asterisk (*) denotes testing for a linear trend. In the multivariable analysis, Model A is adjusted for age and sex, while Model B is adjusted for age, sex, ethnicity, Townsend deprivation index, body mass index, and smoking status. Particulate matter definitions: [$PM_{2.5}$] Finer particles with a diameter less than 2.5 μm; [$PM_{2.5\ absorbance}$] Measures light absorption (blackness) of $PM_{2.5}$ filters, served as a proxy of elemental carbon typically emitted from combustion sources; [PM_{10}] Particles with a diameter of 10 μm or less; [$PM_{2.5-10}$] Coarse particulate fraction between 2.5 μm and 10 μm in diameter.

PM = particulate matter; HR = hazard ratio; CI = confidence interval; IQR = interquartile range; SD = standard deviation; μg/m^3 = microgram per cubic meter; NO_2 = nitrogen dioxide; NO_x = nitrogen oxides.
