# Supplementary material for: Association of Ambient Air Pollution Exposure With Incident Glaucoma: 12-Year Evidence From the UK Biobank Cohort
Source: Invest Ophthalmol Vis Sci. 2024 Oct 16;65(12):22. doi: 10.1167/iovs.65.12.22 (PMC11488522; doi:10.1167/iovs.65.12.22)
Supplement: Supplement 3 [file iovs-65-12-22_s003.pdf]

Table S2. Association Between Air Pollution and Glaucoma Incidence with further adjustment of Charlson Comorbidity Index

Pollutants	Multivariable Model	
	HR (95% CI)	*P*
**$PM_{2.5}$**		
Continuous, per IQR increase	1.02 (0.99 to 1.05)	0.089
Quartiles		
Q1 (8.17 μg/m^3 to 9.29 μg/m^3)	Reference	
Q2 (9.30 μg/m^3 to 9.93 μg/m^3)	0.98 (0.93 to 1.05)	0.637
Q3 (9.94 μg/m^3 to 10.56 μg/m^3)	1.00 (0.94to 1.07)	0.921
Q4 (10.57 μg/m^3 to 21.31 μg/m^3)	1.09 (1.02 to 1.16)	0.010
P for trend*		0.009
**$PM_{2.5\ absorbance}$**		
Continuous, per IQR increase	1.03 (1.00 to 1.05)	0.039
Quartiles		
Q1 (0.83 μg/m^3 to 1.00 μg/m^3)	Reference	
Q2 (1.01 μg/m^3 to 1.13 μg/m^3)	0.99 (0.94 to 1.05)	0.788
Q3 (1.14 μg/m^3 to 1.30 μg/m^3)	1.02 (0.96 to 1.08)	0.610
Q4 (1.31 μg/m^3 to 4.60 μg/m^3)	1.06 (1.00 to 1.13)	0.067
P for trend*		0.051
**$PM_{2.5-10}$**		
Continuous, per IQR increase	1.00 (0.98 to 1.02)	0.965
Quartiles		
Q1 (5.57 μg/m^3 to 5.84 μg/m^3)	Reference	
Q2 (5.85 μg/m^3 to 6.11 μg/m^3)	1.03 (0.97 to 1.09)	0.381
Q3 (6.12 μg/m^3 to 6.64 μg/m^3)	1.02 (0.96 to 1.08)	0.532
Q4 (6.65 μg/m^3 to 12.82 μg/m^3)	1.02 (0.96 to 1.08)	0.524
P for trend*		0.598
**PM_{10}**		
Continuous, per SD increase	1.00 (0.98 to 1.02)	0.783
Quartiles		
Q1 (11.78 μg/m^3 to 15.25 μg/m^3)	Reference	
Q2 (15.26 μg/m^3 to 16.03 μg/m^3)	0.99 (0.93 to 1.05)	0.704
Q3 (16.04 μg/m^3 to 17.01 μg/m^3)	1.01 (0.95 to 1.07)	0.778
Q4 (17.02 μg/m^3 to 31.39 μg/m^3)	1.02 (0.96 to 1.09)	0.491
P for trend*		0.392

**NO_2**		
Continuous, per IQR increase	1.02 (0.99 to 1.06)	0.119
Quartiles		
Q1 (12.93 μg/m^3 to 21.47 μg/m^3)	Reference	
Q2 (21.48 μg/m^3 to 26.23 μg/m^3)	0.98 (0.93 to 1.04)	0.587
Q3 (26.24 μg/m^3 to 31.25 μg/m^3)	1.01 (0.95 to 1.07)	0.746
Q4 (31.26 μg/m^3 to 108.49 μg/m^3)	1.04 (0.97 to 1.11)	0.271
P for trend*		0.198
**NO_x**		
Continuous, per IQR increase	1.02 (0.99 to 1.04)	0.183
Quartiles		
Q1 (19.74 μg/m^3 to 34.38 μg/m^3)	Reference	
Q2 (34.39 μg/m^3 to 42.41 μg/m^3)	1.02 (0.96 to 1.08)	0.583
Q3 (42.42 μg/m^3 to 50.77 μg/m^3)	1.03 (0.97 to 1.09)	0.359
Q4 (50.78 μg/m^3 to 265.94 μg/m^3)	1.06 (0.99 to 1.13)	0.060
P for trend*		0.059

Asterisk (*) denotes testing for a linear trend. In the multivariable model adjusted for age, sex, ethnicity, Townsend deprivation index, body mass index, smoking status, and Charlson comorbidity index. Particulate matter definitions: [$PM_{2.5}$] Finer particles with a diameter less than 2.5 μm; [$PM_{2.5\ absorbance}$] Measures light absorption (blackness) of $PM_{2.5}$ filters, served as a proxy of elemental carbon typically emitted from combustion sources; [PM_{10}] Particles with a diameter of 10 μm or less; [$PM_{2.5-10}$] Coarse particulate fraction between 2.5 μm and 10 μm in diameter.

PM = particulate matter; NO_2 = nitrogen dioxide; NO_x = nitrogen oxides, HR = hazard ratio; CI = confidence interval; IQR = interquartile range; SD = standard deviation; μg/m^3 = microgram per cubic meter; Q1 = quartile 1; Q2 = quartile 2; Q3 = quartile 3; Q4 = quartiles 4.
