# Supplementary material for: Association of Ambient Air Pollution Exposure With Incident Glaucoma: 12-Year Evidence From the UK Biobank Cohort
Source: Invest Ophthalmol Vis Sci. 2024 Oct 16;65(12):22. doi: 10.1167/iovs.65.12.22 (PMC11488522; doi:10.1167/iovs.65.12.22)
Supplement: Supplement 4 [file iovs-65-12-22_s004.pdf]

Table S3. Gene-environment interaction analysis

| Pollutants, μg/m$^3$ | Multivariable Model B | |
|---|---|---|
| | HR (95% CI) | *P* |
| No. of participants / incident cases | 430257 / 8612 | |
| **$PM_{2.5}$** | | |
| Continuous, per IQR increase | 1.03 (1.00 to 1.07) | 0.026 |
| PRS | 1.65 (1.61 to 1.69) | <0.001 |
| $PM_{2.5}$ × PRS | | 0.479 |
| **$PM_{2.5\ absorbance}$** | | |
| Continuous, per IQR increase | 1.03 (1.00 to 1.06) | 0.052 |
| PRS | 1.63 (1.59 to 1.68) | <0.001 |
| $PM_{2.5\ absorbance}$ × PRS | | 0.448 |
| **$PM_{2.5-10}$** | | |
| Continuous, per IQR increase | 1.00 (0.98 to 1.03) | 0.645 |
| PRS | 1.62 (1.58 to 1.67) | <0.001 |
| $PM_{2.5-10}$ × PRS | | 0.134 |
| **$PM_{10}$** | | |
| Continuous, per SD increase | 1.00 (0.98 to 1.03) | 0.962 |
| PRS | 1.64 (1.61 to 1.69) | <0.001 |
| $PM_{10}$ × PRS | | 0.266 |
| No. of participants / incident cases | 462023 / 8832 | |
| **$NO_2$** | | |
| Continuous, per IQR increase | 1.03 (1.00 to 1.07) | 0.058 |
| PRS | 1.65 (1.61 to 1.69) | <0.001 |
| $NO_2$ × PRS | | 0.632 |
| **$NO_x$** | | |
| Continuous, per IQR increase | 1.02 (1.00 to 1.05) | 0.067 |
| PRS | 1.65 (1.61 to 1.69) | <0.001 |
| $NO_x$ × PRS | | 0.442 |

The multivariable Model B accounted for age, sex, ethnicity, Townsend deprivation index, body mass index, and smoking status. Glaucoma PRS was included as a continuous variable in these models and was standardized with a mean of 0 and a SD of 1 for analyses. Particulate matter definitions: [$PM_{2.5}$] Finer particles with a diameter less than 2.5 μm; [$PM_{2.5\ absorbance}$] Measures light absorption (blackness) of $PM_{2.5}$ filters, served as a proxy of elemental carbon typically emitted from combustion sources; [$PM_{10}$] Particles with a diameter of 10 μm or less; [$PM_{2.5-10}$] Coarse particulate fraction between 2.5 μm and 10 μm in diameter.

PM = particulate matter; HR = hazard ratio; CI = confidence interval; IQR = interquartile range; SD = standard deviation; $\mu g/m^3$ = microgram per cubic meter; $NO_2$ = nitrogen dioxide; $NO_x$ = nitrogen oxides; PRS = polygenic risk score.
