# Supplementary material for: Association of Ambient Air Pollution Exposure With Incident Glaucoma: 12-Year Evidence From the UK Biobank Cohort
Source: Invest Ophthalmol Vis Sci. 2024 Oct 16;65(12):22. doi: 10.1167/iovs.65.12.22 (PMC11488522; doi:10.1167/iovs.65.12.22)
Supplement: Supplement 5 [file iovs-65-12-22_s005.pdf]

Table S4. Spearman's correlation coefficients between air pollutants

Pollutants, $\mu g/m^3$	NO_2	NO_x	PM_{10}	$PM_{2.5}$	$PM_{2.5absorbance}$	$PM_{2.5-10}$
NO_2	1.00	0.91	0.54	0.85	0.84	0.29
NO_x	0.91	1.00	0.55	0.87	0.72	0.29
PM_{10}	0.54	0.55	1.00	0.57	0.56	0.78
$PM_{2.5}$	0.85	0.87	0.57	1.00	0.62	0.28
$PM_{2.5absorbance}$	0.84	0.72	0.56	0.62	1.00	0.45
$PM_{2.5-10}$	0.29	0.29	0.78	0.28	0.45	1.00

Particulate matter definitions: [$PM_{2.5}$] Finer particles with a diameter less than 2.5 μm; [$PM_{2.5\ absorbance}$] Measures light absorption (blackness) of $PM_{2.5}$ filters, served as a proxy of elemental carbon typically emitted from combustion sources; [PM_{10}] Particles with a diameter of 10 μm or less; [$PM_{2.5-10}$] Coarse particulate fraction between 2.5 μm and 10 μm in diameter.

PM = particulate matter; $\mu g/m^3$ = microgram per cubic meter; NO_2 = nitrogen dioxide; NO_x = nitrogen oxides.
